# CGHnormaliter: an iterative strategy to enhance normalization of array CGH data with imbalanced aberrations

**DOI:** 10.1186/1471-2164-10-401

**Published:** 2009-08-26

**Authors:** Bart PP van Houte, Thomas W Binsl, Hannes Hettling, Walter Pirovano, Jaap Heringa

**Affiliations:** 1 Centre for Integrative Bioinformatics VU (IBIVU), VU University Amsterdam, De Boelelaan 1081A, 1081 HV Amsterdam, the Netherlands

## Abstract

**Background:**

Array comparative genomic hybridization (aCGH) is a popular technique for detection of genomic copy number imbalances. These play a critical role in the onset of various types of cancer. In the analysis of aCGH data, normalization is deemed a critical pre-processing step. In general, aCGH normalization approaches are similar to those used for gene expression data, albeit both data-types differ inherently. A particular problem with aCGH data is that imbalanced copy numbers lead to improper normalization using conventional methods.

**Results:**

In this study we present a novel method, called CGHnormaliter, which addresses this issue by means of an iterative normalization procedure. First, provisory balanced copy numbers are identified and subsequently used for normalization. These two steps are then iterated to refine the normalization. We tested our method on three well-studied tumor-related aCGH datasets with experimentally confirmed copy numbers. Results were compared to a conventional normalization approach and two more recent state-of-the-art aCGH normalization strategies. Our findings show that, compared to these three methods, CGHnormaliter yields a higher specificity and precision in terms of identifying the 'true' copy numbers.

**Conclusion:**

We demonstrate that the normalization of aCGH data can be significantly enhanced using an iterative procedure that effectively eliminates the effect of imbalanced copy numbers. This also leads to a more reliable assessment of aberrations. An R-package containing the implementation of CGHnormaliter is available at .

## Background

Array comparative genomic hybridization (aCGH) is an experimental approach used to scan an entire genome for copy number changes at a high resolution [1]. These changes occur particularly in oncogenes where mutations can lead to either gains or losses of genetic material. Consequently, aCGH is a commonly used technique to identify aberrations leading to tumors [2-4]. In aCGH experiments, test and reference DNA samples are labeled with distinct dyes and hybridized to cloned DNA fragments of which the exact genomic location is known. For each DNA region the two-dye intensities are measured by fluorescence from which the corresponding log_2 _intensity ratios (*M*) are calculated. A ratio value close to zero indicates a normal copy number (e.g. two in diploids) while a value above or below zero indicates a gain or a loss, respectively. Nonetheless, the proper assessment of copy numbers is not a trivial task and several computational algorithms have been developed for normalization, smoothing, segmentation and calling [5-9]. The normalization procedure, the first stage of the aCGH analysis, aims to minimize the effect of the technical bias (e.g. dye bias) in log_2 _intensity ratios. Usually aCGH normalization is based upon methods applied to gene expression data, i.e. global-median and intensity-based LOWESS normalization [10]. In global-median normalization a median *M *value is determined and subtracted from all *M *values. By doing so, the *M *values become centered around a median value of zero. Intensity-based LOWESS normalization instead fits a smooth regression line through all *M *value points. Normalization is achieved by subtracting from each *M *value its corresponding regression value. These conventional techniques however are in the majority of cases not applicable to aCGH data. This is due to the fact that relevant biological variation is often erroneously treated as technical bias and removed. For instance, probes corresponding to gains (which on average have higher intensities) are generally 'over-normalized' making a proper assessment of gains more difficult. A recently developed method, called popLowess, attempts to tackle this problem by separating the aberrations from the normals through k-means (*k *= 3) clustering [11]. In this manner the normalization is only based on the population of normal probe values. The problem however is that 'calling' through a clustering method is rather course-grained while several more refined methods are available [9,12-14]. Another recent normalization and centralization method that seeks to overcome over-normalization was proposed by Chen *et al*. [5]. In their algorithm normalization is performed by regressing the highest ridgeline of a 2-dimensional intensity distribution which is assumed to correspond to normal probes. Subsequently, the most occurring probe intensity (i.e. the highest peak in the intensity distribution) is used for centralization.

**Figure 1:**
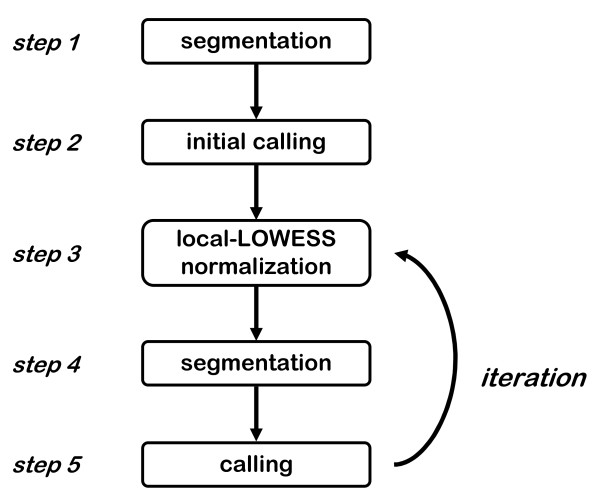
**Overview of the CGHnormaliter method**. First break-points and segments are detected (step 1) and initial calling is performed to distinguish normals from gains and losses (step 2). Subsequently, a 'local-LOWESS normalization' procedure is followed which uses only the normals to calculate the LOWESS regression curve (step 3). The following step consists of break-point detection and segmentation (step 4) after which new calls are established (step 5). If the latter calling differs with respect to the previous calling, the procedure is repeated from step 3.

In this study we present a new method, called CGHnormaliter, which offers a more sophisticated normalization of aCGH data. First we perform segmentation after which we use a custom calling method to accurately assess normals, gains and losses. In the 3^rd ^step we fit a LOWESS regression curve through the normals only and use that to normalize the entire population of probes. Subsequently new segments are determined. In step 5 we re-run the calling method which can reach an even higher calling accuracy based on the normalized data. Steps 3–5 are then iteratively repeated until convergence or a user-specified maximum number of iterations is reached. An overview is given in Figure 1. We applied our method to three tumor-related aCGH datasets for which the 'true' copy number changes were determined using spectral karyotyping (SKY), G-banding and/or fluorescence in situ hybridization (FISH). To the best of our knowledge such an extended and high quality benchmark is used here for the first time. The performance was compared to three other normalization strategies: a conventional global-median approach, popLowess and the method by Chen *et al*. In the majority of cases CGHnormaliter showed a higher performance in terms of sensitivity, specificity and precision. An implementation of CGHnormaliter is available as an R-package and can be downloaded from .

## Results and Discussion

### Evaluation criteria

In this work we compared the performance of three previously proposed normalization strategies (global-median, popLowess and Chen *et al*.) to our novel CGHnormaliter method. Our benchmark consists of three aCGH datasets with experimentally verified imbalanced aberrations. Normalization methods were applied to each benchmark set, followed by segmentation and calling using DNAcopy [15] and CGHcall [14], respectively. Subsequently the calling performance was evaluated in terms of sensitivity, specificity and precision. Sensitivity is calculated as follows:



where *TP *indicates the number of true positives and *FN *the number of false negatives. Specificity is defined as:



where *TN *indicates the number of true negatives and *FP *the number of false positives. Finally, precision is defined as:



Our interpretation of *TP*, *TN*, *FP *and *FN *is explained in Table 1. As a standard procedure, we have taken the gains and losses as positives since they are indicative for disease.

**Table 1:** Definition of true positives (TP), false positives (FP), true negatives (TN) and false negatives (FN).

		prediction (call)	
		gain/loss	normal

gold	gain/loss	TP	FN
	
standard	normal	FP	TN

### Performance on the acute lymphoblastic leukemia (ALL) dataset

**Figure 2:**
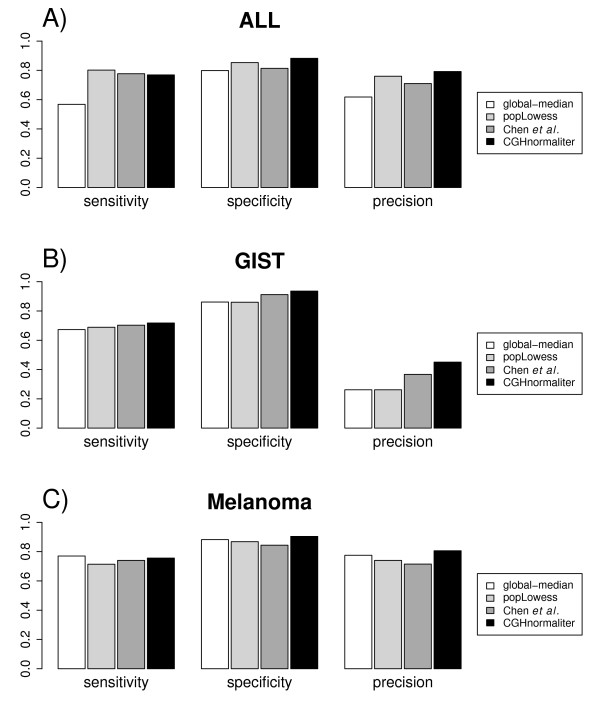
**Comparison of method performance in terms of sensitivity, specificity and precision**. Overall results for **(A) **the acute lymphoblastic leukemia (ALL), **(B) **the gastrointestinal stromal tumor (GIST), and **(C) **the human melanoma cell lines dataset are given.

**Figure 3:**
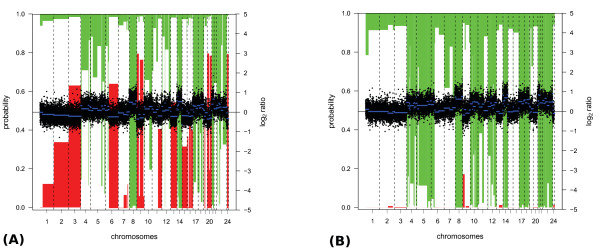
**Example of the effects over-normalization using global-median normalization**. Calling results on an ALL tumor sample (sample 4) after **(A) **global-median and **(B) **CGHnormaliter normalization are shown. In these figures normalized log_2 _intensity ratios and segments are represented by dots and blue horizontal lines, respectively. Aberration probabilities are indicated by the length of the green downward (gain) and red upward (loss) bars. Note that segments are designated gain or loss if their probabilities exceed 0.5. G-banding and FISH analyses revealed gains in 14 chromosomes (4, 5, 6, 7, 8, 10, 11, 12, 14, 17, 18, 21, 22, 23(X)) most of which are confirmed using CGHnormaliter. Over-normalization caused by global-median normalization instead leads to many incorrect calls.

In Figure 2A the average performance of all methods on the ALL dataset is displayed. From this figure it is clear that global-median normalization is outperformed by all other methods. popLowess and CGHnormaliter yield comparable results for all evaluation criteria (0.81 on average). Chen *et al*. performs slightly worse (0.77 on average) whereas global-median scores are considerably lower for sensitivity (0.57) and precision (0.62). We also investigated the underlying causes of the inferior performance of global-median normalization. As expected we found, particularly in cases where a large number of imbalanced aberrations occur, that global-median does not properly yield a normal copy number. In such cases, 'over-normalization' of the data occurs, leading to excessively shifted spot intensity ratios. A salient example is given in Figure 3, where calling results of a tumor sample are shown after global-median and CGHnormaliter normalization. In this sample gains were experimentally verified in 14 out of 24 chromosomes. In the global-median approach the median is rather high, leading to an overestimation of the number of losses and underestimation of the number of gains. In fact, only 11 out of 14 gains were (partially) recognized. CGHnormaliter (and also popLowess) attempts to correct for this problem and is able to properly identify 13 gains. Finally, in Table 2 we compare the effect of each normalization method on the resulting *M *values. It is clear that alternative strategies lead to considerably different shifts in the *M *values, whereas the final calling results are more similar (see Figure 2A).

**Table 2:** Effect of different normalization strategies on the *M *values.

	ALL	GIST	Melanoma
global-median	-0.098	0.344	-0.751
popLowess	-0.239	0.348	-0.760
Chen *et al*.	-0.228	0.315	-0.508
CGHnormaliter	-0.204	0.304	-0.439

Numbers represent the mean shifts between the original and normalized *M *values.

### Performance on the gastrointestinal stromal tumor (GIST) dataset

**Figure 4:**
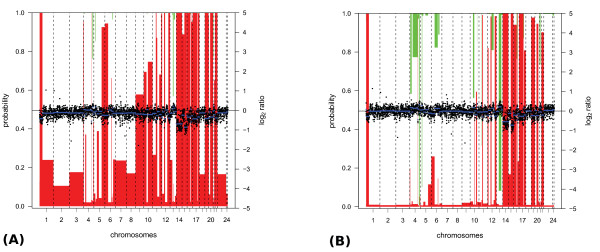
**Example where CGHnormaliter achieves better results than popLowess due to more sophisticated clustering of the intensity ratios**. Calling results on a GIST tumor sample (sample 57) after **(A) **popLowess and **(B) **CGHnormaliter normalization are shown. FISH analyses revealed losses in 2 chromosomes (14 and 15). All losses are identified largely by both methods but CGHnormaliter scores better in specificity and precision. The figure format is explained under Figure 3.

Results on the GIST dataset are summarized in Figure 2B. CGHnormaliter performs best on all evaluation criteria (sensitivity = 0.72, specificity = 0.94 and precision = 0.45). The method by Chen *et al*. is second best and scores 4 percentage points lower on average. Furthermore global-median and popLowess show similar performances but on average 10 percentage points lower than CGHnormaliter. In Figure 4 we further elucidate the differences in performance between popLowess and CGHnormaliter. For this tumor sample, losses were experimentally verified in 2 out of 24 chromosomes. Although both methods are able to identify a considerable part of them, CGHnormaliter scores considerably better in specificity and precision (plus 8 and 19 percentage points, respectively). This can be explained by the observation that popLowess identifies only a fraction of the 'true' normals as normals during its clustering step, so only normals with high *M *values are used for normalization. As a consequence the LOWESS regression line becomes too high and the normalized *M *values too low, leading to an overestimation of the number of losses. In CGHnormaliter the normals are better recognized, yielding a more accurate centralization of the *M *values. The difference between normalization methods in terms of the resulting *M *values is substantial though less pronounced relative to the ALL dataset (Table 2).

### Performance on the human melanoma cell line dataset

Results on the melanoma dataset are shown in Figure 2C. CGHnormaliter performs best on specificity (0.90) and precision (0.81), while global-median normalization is slightly more sensitive than CGHnormaliter (0.77 versus 0.76). popLowess and Chen *et al*. perform several percentage points worse compared with CGHnormaliter on all evaluation criteria. It should be stressed however that the somewhat higher sensitivity yielded by global-median can be attributed to a strikingly good performance on a single sample being the human melanoma cell line WM983. In this case centralization of aCGH data is rather complicated since more than half of the WM983 genome is aberrated. Overall the results are in line with the previous two datasets where CGHnormaliter outperforms the competitors tested.

### Perspectives/limitations

The work we present here is based on a thorough comparison of a number of aCGH normalization methods involving several testsets. Further investigation should not only comprise larger datasets containing significantly more samples, but should also involve additional high-density platforms, such as Nimblegen. It goes without saying that future development of aCGH data analysis methods will be largely dependent on the size and quality of benchmark sets.

As a next step in the future development of our method we aim to extend the protocol by allowing single-channel data, or dual-channel data for which intensity values are not available. This could be achieved by implementing an iterative local-median strategy as an alternative to the local-LOWESS strategy currently used. In this way the general applicability of CGHnormaliter would be enhanced.

Finally, it should be stressed that a major pitfall of all methods occurs in cases that display many imbalanced copy number alterations. In samples where the number of gains or losses exceeds the number of normals, the data will be centralized around these gains or losses, leading to an incorrect normalization. Another drawback appears in sets where the ploidy of the reference and test sample differs, usually as a result of hypoploidy of the test sample. For instance, if the ploidy of reference and test are *m *and *n *(where *m *≠ *n*), respectively, the centralization should be around  instead of zero as employed by current methods. The integration of prior knowledge concerning the ploidy, number and nature of aberrations is likely to be key in alleviating these complications.

## Conclusion

We introduce a new strategy, called CGHnormaliter, for improved normalization of aCGH data displaying imbalanced aberrations. Our method was tested on three well-studied test sets (ALL, GIST and Melanoma) which are unique considering the large number of extensively validated samples and the occurrence of many imbalanced aberrations. The performance was compared with a conventional global-median approach and the recently published tools popLowess [11] and that by Chen *et al*. [5]. We conclude that on average CGHnormaliter outperforms the three other methods in terms of specificity and precision, while its overall sensitivity is comparable to that obtained by popLowess and Chen *et al*.. The global-median approach scores considerably lower on almost all data samples, mainly due to over-normalization: the presence of many imbalanced aberrations leads to an improper centralization of the intensity ratios. Furthermore, in a number of cases popLowess and Chen *et al*. achieve similar results as CGHnormaliter since all methods only use the normals for normalization. However, in some examples the identification of the normals is not trivial. In such cases the iterative refinement steps of CGHnormaliter yield better results than the single clustering step of popLowess or the 'highest ridgeline regression' strategy by Chen *et al*.. It would be interesting to further investigate these findings and combine the iterative protocol with alternative normalization approaches. Nonetheless this research emphasizes the importance of normalization based on properly defined normals and shows the added value of iteration for proper assessment of such normals.

## Methods

### CGHnormaliter algorithm

CGHnormaliter is a normalization method tailored to aCGH data. Its novelty resides both in the fact that normalization is guided by a more sophisticated calling technique and that further refinement is attained through a new iteration procedure. The strategy can be summarized as follows. Initially the log_2 _intensity ratios are segmented using DNAcopy [15]. The segmented data are then given as input to a recently developed calling tool named CGHcall [14] to discriminate the normals from gains and losses. The assumption here is that the temporary exclusion of aberrations allows for a more appropriate calculation of the LOWESS regression curve. As a result, after normalization, the log_2 _intensity ratios of the normals will generally be closer to zero and better reflect the biological reality. We coin this normalization strategy 'local-LOWESS' because only a subset of the intensity ratios is considered in the LOWESS regression. The thus normalized data are then segmented again and called. It is likely that the new calls will be more accurate than the previous ones because these are now based on normalized data. In turn, further iterative normalization might benefit from these improved calls. To control iteration, CGHnormaliter needs to establish whether the normalization results have been significantly changed or not. Iterations are terminated if each of the samples shows a mean difference relative to its value in the preceding iteration below α (default α = 0.01). Alternatively, the user can set a maximum number of iterations.

We also included a feature to prevent 'wandering' of the median during the iterative steps of CGHnormaliter. This might occur if a large number of gains or losses are present. In this situation it is likely that the calling algorithm will select many of these as normals. As a consequence an undesired upward (or downward) bias of the baseline can be observed, resulting in a biologically unrealistic number of losses (or gains), which will typically get worse during subsequent iterations. To prevent this we denote the largest copy number population as normals and adjust all calls accordingly.

### Other normalization methods

To test the global-median normalization strategy, we used the implementation in the R-package CGHcall version 1.2.0 [14]. In this routine standard global-median normalization is combined with a smoothing step [15] to remove outliers. For popLowess we used the standalone version 1.0.1 (with a lower limit of 1 for the 'smoothing size' to guarantee normalization of all chromosomes). For the method by Chen *et al*. we used the MatLab implementation provided by the authors. All programs were run using default parameter settings.

### Data

In this study we used three tumor-related benchmark aCGH datasets for method evaluation. These were selected since they contain a considerable amount of samples with imbalanced copy numbers which are cytogenetically verified using SKY, G-banding and/or FISH. The first dataset comprises 8 acute lymphoblastic leukemia (ALL) tissue samples which were analyzed using 32 K BAC arrays ([16], see Additional file 1). The second dataset consists of 17 gastrointestinal stromal tumors (GIST). These were analyzed using 3 K BAC and PAC arrays where only spots with signal intensities of at least two times the background intensities were included ([17], GSE5336, see Additional file 2). The third dataset includes samples from 4 human melanoma cell lines, which were analyzed using Agilent 44 K oligonucleotide-based CGH arrays ([18], GSE7822, see Additional file 3).

## Availability and requirements

Project name: CGHnormaliter R package

Project home page: 

Operating system(s): Platform independent

Programming language: R

Licence: GNU GPLv3

## Authors' contributions

All authors were involved in the design of this study. TWB, BvH and HH performed the research. BvH, WP and JH wrote the manuscript. All authors read and approved the final manuscript.

## Supplementary Material

Additional file 1 **Array CGH data of lymphoblastic leukemia (ALL)**. Dataset containing the raw intensities of 8 ALL samples analyzed using 32 K BAC arrays [16]. Click here for file

Additional file 2 **Array CGH data of gastrointestinal stromal tumors (GIST)**. Dataset containing the raw intensities of 17 GIST samples analyzed using 3 K BAC and PAC arrays [17]. Click here for file

Additional file 3 **Array CGH data of human melanoma cell lines**. Dataset containing the raw intensities of 4 human melanoma cell lines analyzed using Agilent 44 K oligonucleotide-based CGH arrays [18]. Click here for file
